# The space charge limited current and huge linear magnetoresistance in silicon

**DOI:** 10.1038/s41598-017-19022-1

**Published:** 2018-01-15

**Authors:** Y. Liu, H. Wang, X. Jin, M. Zhang

**Affiliations:** 1 Department of Physics, Capital Normal University, Beijing, 100048 P.R. China; 2 Beijing Key Laboratory of Metamaterials and Devices, Beijing, 100048 P.R. China; 3 Key Laboratory of Terahertz Optoelectronics, Ministry of Education, Beijing, 100048 P.R. China; 4 Beijing Advanced Innovation Center for Imaging Technology, Beijing, 100048 P.R. China

## Abstract

Huge magnetoresistance in space charge regime attracts broad interest on non-equilibrium carrier transport under high electric field. However, the accurate fitting for the current-voltage curves from Ohmic to space charge regime under magnetic fields has not been achieved quantitatively. We conjecture that the localized intensive charge dynamic should be taken into consideration. Here, by introducing a field-dependent dielectric constant, for the first time, we successfully simulate the current-voltage curves of covalent crystal silicon wafers under different magnetic fields (0–1 Tesla). The simulation reveals that the optical phonon, instead of the acoustic phonon, plays a major role for the carriers transport under magnetic fields in space charge regime.

## Introduction

The space charge effect covers vast applications, such as solar cells^1,2^, light-emitting diodes^3^, electro-resistance^4,5^ and high-power semiconductor devices^6^. The theoretical approach on the effect can be traced back to 1950s. Earlier models postulated the density state distribution of band tail, either Gaussian or exponential, for matching experiment data^7–9^. Recently, it has been revealed that the interplay between dopants and traps^10^, or molecular sites and the free-carrier density of states^11,12^, controls current-voltage (*I-V*) characteristics in semiconductors.

Remarkably, in 2009, Delmo *et* *al*. achieved a large positive magnetoresistance of more than 1,000 percent in silicon at room temperature that could be explained by the quasi-neutrality breaking of the space-charge effect^13^. The phenomena may originate from a spatial inhomogeneous electric field or an inhomogeneous charge distribution^13,14^. Experimentally, the magnetoresistance can be controlled by the current, the magnetic field^15^, the dopant concentration^16^, the electrode configuration^14,17^ and the difference of the surface/bulk electron combination rate^18^. One could predict a non-saturating huge linear magnetoresistance through a macroscopic random resistor network model^19,20^ or evaluate the net-charge distributions via finite element calculations^14,17^. However, the relationship between the spatial charge dynamic and the large linear magnetoresistance effect remains obscure.

Here, we consider that under a high electric field, the localized charges are forced to be itinerary; meanwhile, the itinerant charges can be captured by the ion cores. The dynamic of ionization and filling results in a high-energy electron-phonon interaction, which emits (or annihilates) virtual phonons and enhances (or reduces) the local carrier density^10,11,21^. By introducing a field-dependent dielectric constant, we successfully simulate the *I-V* curves of silicon wafers from Ohmic to space-charge regimes under different magnetic fields based on the theorem proposed by Zhang and Pantelides (ZP model). A positive linear magnetoresistance over 2,600% is obtained for an intrinsic N-Si in the space-charge regime under 1.2 T at room temperature. The capacitance of the intrinsic N-Si quasi-linearly increases under applied voltage, but shows a quadratic decrease under magnetic field. Although a heavily-doped N-Si or a P-Si possesses a quite similar *I-V* curve with an intrinsic N-Si, their magnetoresistances are negligible at room temperature.

## Experimental details

The double-sides polished silicon wafers (thickness, 0.5 mm; Ke Jing Materials Technology, HeFei) with different doping type and resistance were used. Naturally oxidized layer on silicon wafer was removed by hydrofluoric acid. Then good ohmic indium/silicon contacts were fabricated under 400 °C for ten minutes. The two-terminal magnetotransport was measured using a source meter (Keithley 4200) under a EMP-7 electromagnet (East Changing Technology, Beijing) at room temperature. The capacitances were measured by an Andeen-Hagerling 2700 A ultra precision capacitance bridge with 10 V alternating excitation signal under different the bias voltage and magnetic field. It needs to be emphasized that the indium electrodes were pressed on center of the upper and lower surfaces of the silicon wafer. The lateral size of silicon wafer was not less than 16 mm, therefore, the distance between the contacts *L* and the width *W* meets *L* ≪ *W*.

**Figure 1:**
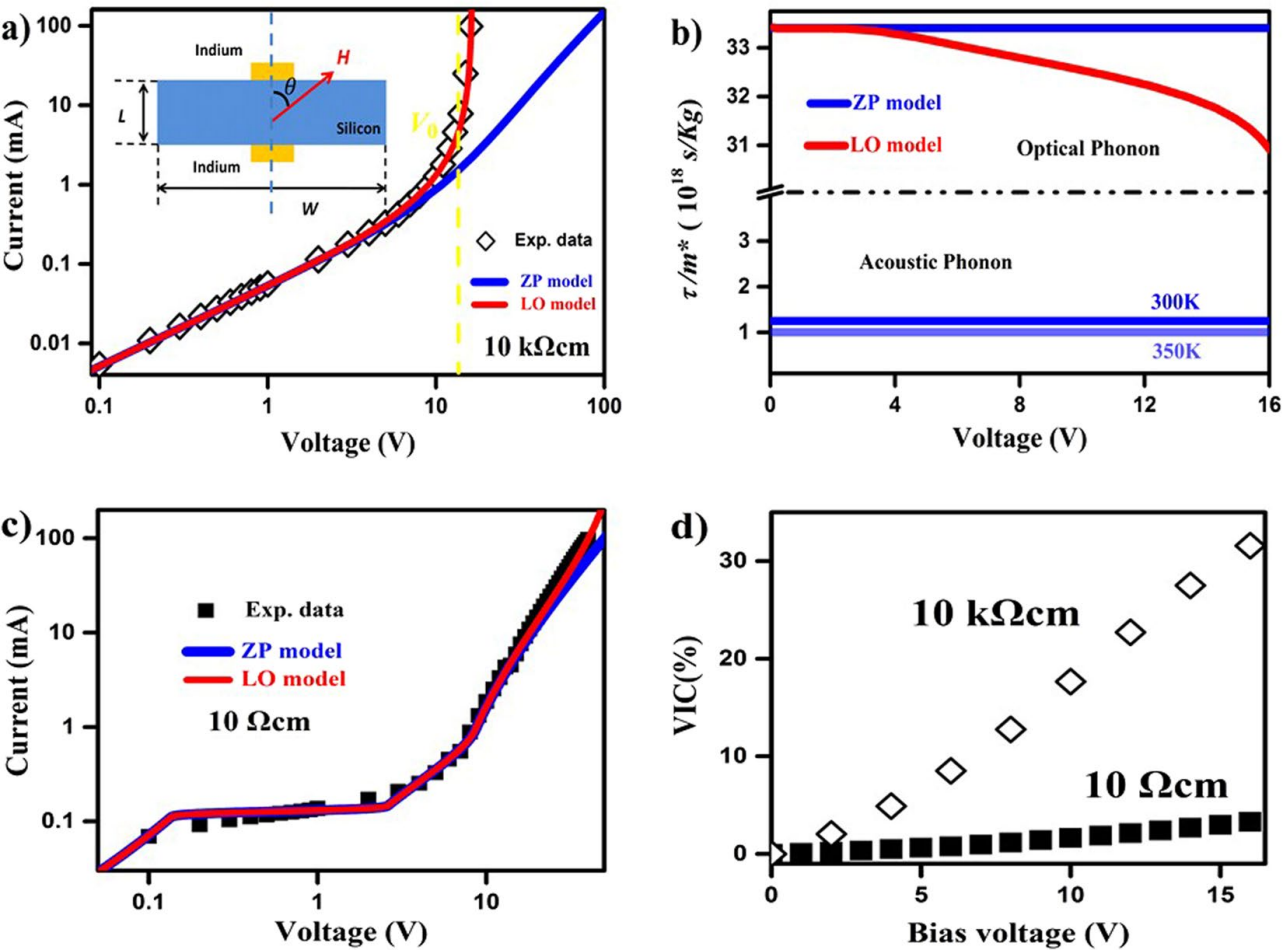
Fits for *I-V* curves of the intrinsic and heavily doped N-Si without applying magnetic field and the capacitance measurements. (**a**) The *I-V* curve for an intrinsic N-Si with nominal resistivity >10 *K*Ω·*cm* is measured at room temperature plotted on a double logarithmic scale. The blue solid line is the prediction by ZP model and the red solid line is the simulation by the LO model. The critical voltage *V*_0_ is the crossover value by tangent lines of Ohmic and space-charge regimes. (Inset) This device is fabricated on the 16 mm × 16 mm substrate of thickness *L* = 0.5 mm, and the indium contacts are about 1 mm × 1 mm. The indium electrodes are pressed on the center of the upper and lower surfaces of the silicon wafer. *θ* is defined as the angle between the transport and the applied magnetic field direction; (**b**) *τ*/m* of the acoustic phonon and LO phonon branches are evaluated by ZP model and LO model, respectively. (**c**) The *I-V* curve for a heavily doped N-Si with nominal resistivity 10 Ω·*cm* measured at room temperature is plotted on a double logarithmic scale. The blue solid line is the prediction by ZP model and the red solid line is the simulation by the LO model. (**d**) *VICs* versus bias voltage, where the open diamonds and the solid squares represent the intrinsic and the heavily doped N-Si, respectively.

## Result and Discussion

As shown in Fig. 1a, a typical *I-V* curve of an intrinsic N-Si exhibits a slow rise (*V* < 10 *V*) followed by a sharp, power-law rise at a critical voltage *V*_0_ (black open diamonds). In order to avoid the competition of the surface and bulk parallel paths^18^ and the edge effects of a limited-size sample^14^, a symmetric out-of-plane electrode set-up is selected in experiments, as shown in the insert of Fig. 1a. The slow rise is the Ohmic regime, and the sharp rise belongs to the space-charge regime according to space-charge-limited-currents theory^7–10^. Recently, Zhang and Pantelides evidenced in theory and experiment that the interplay between dopants and traps controls the power-law rise of *I-V* curves^10^. The prediction of ZP model is drawn in Fig. 1a (blue solid line) with the calculation parameters: the dielectric constant *ε*_*r*_ = 12^22^, dopant density *N*_*D*_ = 1.2 × 10^17^ *m*^−3^, and trap density *N*_*t*_ = 2 × 10^17^ *m*^−3^ ^16^. It is clear that the predicted current by ZP model significantly lags behind the experiment data at high electric field. In principle, for a high purity semiconductor, the acoustic and optical phonon scatterings dominate the transport of carriers, while the ionized impurity contribution can be neglected at room temperature^22^. In respect to the matters, as presented in Fig. 1b, the ratios of the relaxation time and the effective mass of carrier *τ*/m* with the external electric fields are evaluated by ZP model for both the optical and the acoustic phonons at room temperature (blue lines in Fig. 1b). Obviously, *τ*/m* of the optical and acoustic phonons remain a constant with the increase of voltage in ZP model. The acoustic phonon *τ*_*a*_/*m*_a_* obeys a power law rise of temperature in a broad temperature range rather than a function of electric field^10,22^. The higher the temperature is, the lower the carrier mobility is, which is homogenous in sample and offers a minor correction for carrier transport if Joule heat is limited.

ZP model may underestimate the growth of carrier density due to intense electro-phonon excitation. We conjecture that the overall dielectric constant *ε*_*r−sum*_ can be expressed as 1 + *χ* + *χ*_*n*_ + *χ*_*L*_, where *χ*, *χ*_*n*_ and *χ*_*L*_ are the intrinsic, the non-equilibrium and the orientation terms, respectively. *χ*_*n*_ is assumed proportional to the density of non-equilibrium carriers. *χ*_*n*_ = *η*Δ*n/n*_0_, here Δ*n* defines as the density of non-equilibrium carriers, *n*_0_ is the density of thermal-excited carriers and *η* is an arbitrary parameter for simulation. *χ*_*L*_ can be expressed as *γ*ln(1 + Δ*n/n*_0_) with a fitting coefficient *γ*. Under a high electric field, the uncompensated charge and the concomitant push-back electrostatic field yield the spatial distribution of polarity-conserved charges^14,17,18^. The localized charges are polarized along electric field, and the term of polarization convergence ought to be considered even for a non-polar one. For our experiments, a symmetric out-of-plane electrode set-up with *L* ≪ *W*, thus the non-uniform electric field in silicon is fusiform with rotational symmetry. The higher the electric field applies, the stronger convergence of the polarization is. Therefore, we introduce a modified *Langevin* function^23^ in the framework of Mott-Gurney theorem to describe the polaron inhomogeneity under fields (Supplemental Material S1).

Using *ε*_*r−sum*_ in ZP model, the *I-V* curve can be successfully simulated in both Ohmic and space-charge regimes for the intrinsic N-Si, as indicated in Fig. 1a (red solid line). It is noted that the corresponding *τ*_*o*_/*m*_o_* of LO phonon decreases significantly with the applied voltage at non-Ohmic regime, as demonstrated in Fig. 1b (red solid line), which is obviously different from unchanged LO phonon predicted by ZP model (blue solid line). The decrease of *τ*_*o*_/*m*_o_* refers to the enhancement of the localized charge dynamic under high fields. The modified ZP model by field-dependent *ε*_*r−sum*_ is named as LO model to emphasize phonon contribution.

In the case of a heavily-doped N-Si, the thermal excited carriers dominate its properties. The localized high-energy processes are screened by the strong Coulomb effect and the contribution of LO phonons is therefore negligible^21^. It is understood that PPV, polycrystal SrBi_2_Ta_2_O_9_ and nanocrystal CdS thin films, with relative larger density of dopants and traps, can be simulated by ZP model^10,24^. As presented in Fig. 1c, the *I-V* curve of a heavily-doped N-Si exhibits a slow rise followed by a power-law rise at a critical voltage *V*_0_, with the Mott-Gurney limit attained asymptotically (black solid squares)^7–10^. ZP model (blue solid line) fits well with the experiment data as V < 10 V, but the fitting curve slightly lags behind the current increase at high electric field. In principle, the Coulomb screening in heavily-doped N-Si effectively suppresses the emission and trapping of a charge between a lattice site and the free-carrier states. The field-dependent electro-LO phonon interaction compensates the current increase at high electric field, resulting in a better simulation (red solid line). Here, the influence of the electrode configuration is degraded significantly as the sample behaves like a conductor.

Under a high electric field, the virtual phonon emissions are accompanied with the lattice distortions, which alter the charge distributions. The uncompensated charge and the concomitant push-back electrostatic field yield the polarity-conserved charges accumulated on boundaries^10,14^. It is no doubt that the capacitance of sample is modified accordingly. The voltage-induced capacitance is defined as *VIC* = (*C*_*V*_/*C*_0_ − 1) × 100%, where *C*_0_ and *C*_*V*_ are the capacitances under zero and a certain voltage. As shown in Fig. 1d, *VICs* of both an intrinsic N-Si (black open diamonds) and a heavily doped N-Si (black solid squares) show quasi-linear growth with the increase of the bias voltage. Whereas, for a heavily doped N-Si, *VIC* has only a ~4% increase from 0 to 16 V (black solid squares), which is much smaller than the ~30% increase in an intrinsic N-Si. Although the spatial dynamic of ionization and filling occurs at a lattice site, the strong electron-LO phonon interaction accompanied with the virtual phonon processes modifies the dielectric responses of sample. Approaching to a metallic limitation, such as a heavily doped one, the dynamic gets weakened significantly and the intense electron-LO phonon interaction is forbidden^21^.

**Figure 2:**
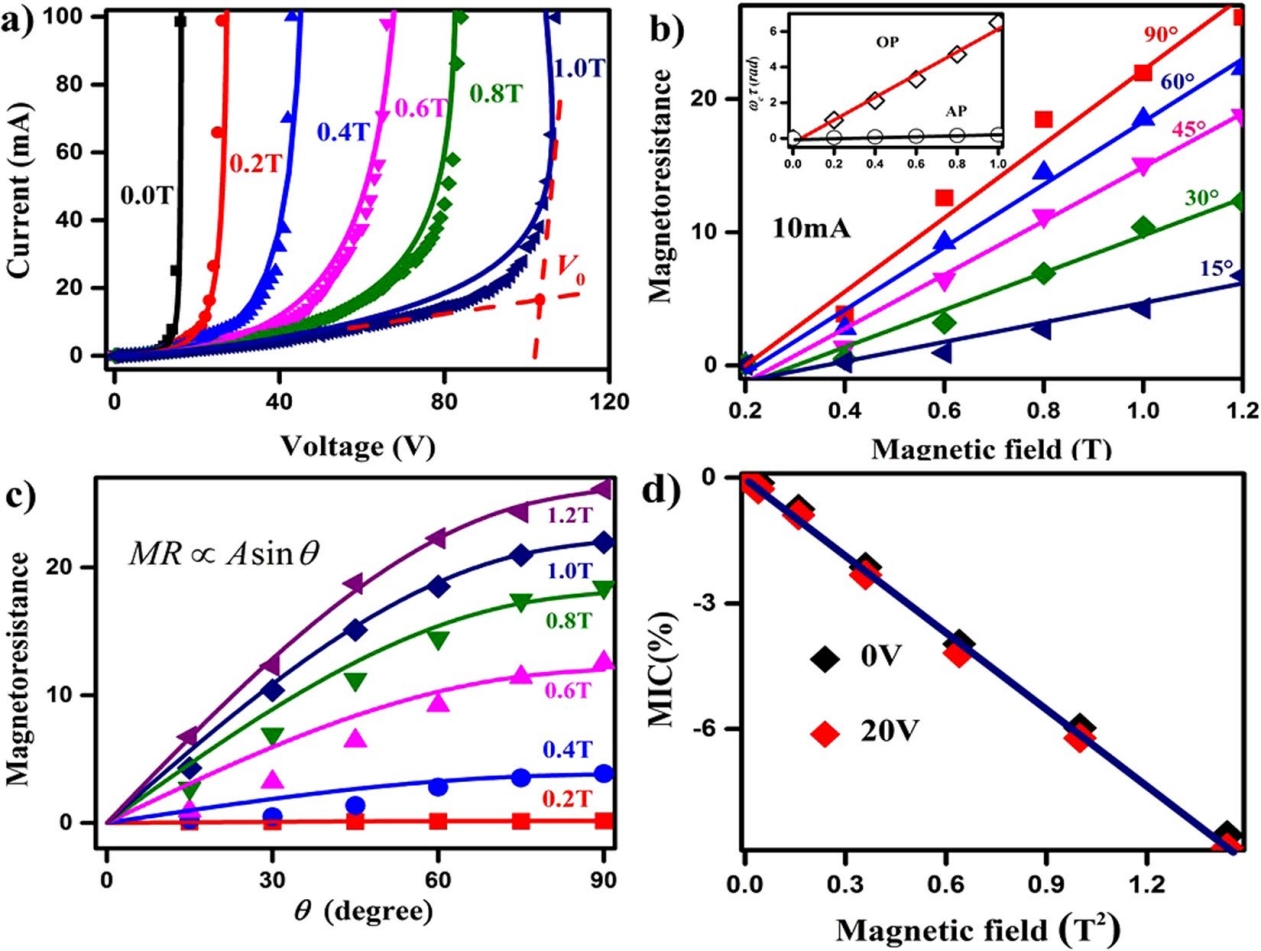
Fits for the I-V curves under magnetic field of the intrinsic N-Si and the magnetoresistance response. (**a**) Experiment data (scatters) are fitted using the LO model (solid lines) with the magnetic field ranging from 0 to 1 T. *V*_0_ shows a linear growth from 14 to 104 V. (**b**) The magnetoresistance is measured at a constant current mode *I* = 10 *mA* under different *θ*. Insert, *ω*_*c*_*τ* of optical and acoustic phonons are shown by the open diamonds and circles, respectively. The red and the black lines are fitted by eyes. (**c**) The relationship between magnetoresistance and *θ* follows *A*sin*θ* under different magnetic fields. (**d**) *MIC* versus the square of the magnetic field under 0 and 20 V are shown as black and red solid diamonds, respectively. The linear fitting is guided by eyes.

Figure 2a shows the typical *I-V* curves for an intrinsic N-Si measured at different magnetic fields. Here, the magnetic field is parallel to the sample surface and perpendicular to the transport direction, as illustrated in the insert plot of Fig. 1a. The *I-V* curve is more sensitive to the magnetic field in the space-charge regime than in the Ohmic regime. The magnetoresistance approaches to zero in Ohmic regime, which is exactly consistent with the classical equilibrium magneto-electric transport theory^21^. The turn-on voltage, *V*_0_, grows quasi linearly, from 14 to 104 V, with the increase of magnetic field from 0 to 1 T. Under a magnetic field, the current suppression constrains the field-induced ionization along the transport direction^13,18,25^, resulting in the postponed of *V*_0_. In fact, although such phenomena are reported in those pioneer works for silicon wafers^13,15,17,26^, to our best knowledge qualified explanations have not been achieved. Here, we present that I-V curves under different magnetic fields are successfully simulated by LO model with a set of unified fundamental parameters, such as *N*_*D*_, *N*_*t*_ and energy levels *etc*., as presented in Fig. 2a by the solid lines (Supplemental Material S3).

The magnetoresistance is defined as [*ρ*(*H*)/*ρ*(0) − 1] × 100%, with *ρ*(0) and *ρ*(*H*) the resistance at zero and applied magnetic field, respectively. In Fig. 2b, the out-of-plane magnetoresistances measured at a constant current mode *I* = 10 *mA* under different *θ* are shown together. The measurements are performed below the breakdown voltages to ensure the data stability. The inhomogeneous spatial dynamic of ionization and filling is inevitably influenced by a perpendicular magnetic field under a certain voltage, viz., prompting the traverse filling and suppressing the ionization along carrier transport direction^21,25^. In our experiment, the magnetoresistance has an excellent linear relationship with magnetic fields under different *θ*, as shown in Fig. 2b, exhibiting a pronounced anisotropic behavior. As *θ* = 90°, the magnetoresistance reaches ~2600% at 1.2 T, which is much larger than those reported in the former works^13,15,26^. In the case of *θ *= 15°, the magnetoresistance is ~400% under 1.2 T, which is an order of magnitude smaller than the one at *θ* = 90°.

Numerous theories implicate spatial variation of the carrier mobility as being responsible for such an anomalously huge magnetoresistance^19,20,27–30^. The spatial variation can be aroused by several factors, such as macroscopic inclusions^31^, geometric configurations^15,17,18^, defects^16,27,32^, and electric field fluctuations^13–15,18,25,26^ in nonmagnetic materials. The magneto-induced phonon resonance relays on the inelastic inter-Landau-level scatterings^33^. It is known that the value of the inhomogeneous magnetoresistance follows Δ*ρ/ρ* ∝ *ω*_*c*_*τ*^27,28^, where *ω*_*c*_ is the cycling frequency and *τ* is the relaxation time of carriers. As shown in the inserted plot of Fig. 2b, *ω*_*co*_*τ*_*o*_ of optical phonon shows a linear increase with a 600% change as magnetic field ≤1T. Whereas, the *ω*_*ca*_*τ*_*a*_ of the acoustic phonon also exhibits a linear increase under magnetic field with only a 20% change. At Ohmic regime, the mobility of carriers is uniform, and the secondary deflection current of Hall electric field offsets that of Lorenz force. But the balance breaks due to the excitation of high-energy electrons at the space-charge regime^21^. As demonstrated in Fig. 2c, the magnetoresistance grows nonlinearly with the increase of *θ* and reaches the maximum as *θ* = 90°. The curves of the magnetoresistance and *θ* can be fitted well by *A*sin*θ* with the arbitrary coefficients.

The magnetic-field-induced capacitance (*MIC*) variations are evaluated experimentally. *MIC* is defined as *MIC* = (*C*_*H*_/*C*_0_ − 1) × 100%, where *C*_0_ and *C*_*H*_ are the capacitances at zero and a certain magnetic field, as shown in Fig. 2d. *C*_0_ are 59 pF and 81 pF for 0 V and 20 V bias voltages, respectively. Although their absolute values are different, their *MICs* have a quite similar magnetic field dependence, *MIC* ∝ −*H*^2^. The quadratic magnetic field dependence is in line with the classical electromagnetism theory for the dielectric response under magnetic field. A perpendicular magnetic field regulates the inhomogeneity and reduces of LO phonon scattering along carrier transport direction, which subdues the dielectric response along transport direction.

**Figure 3:**
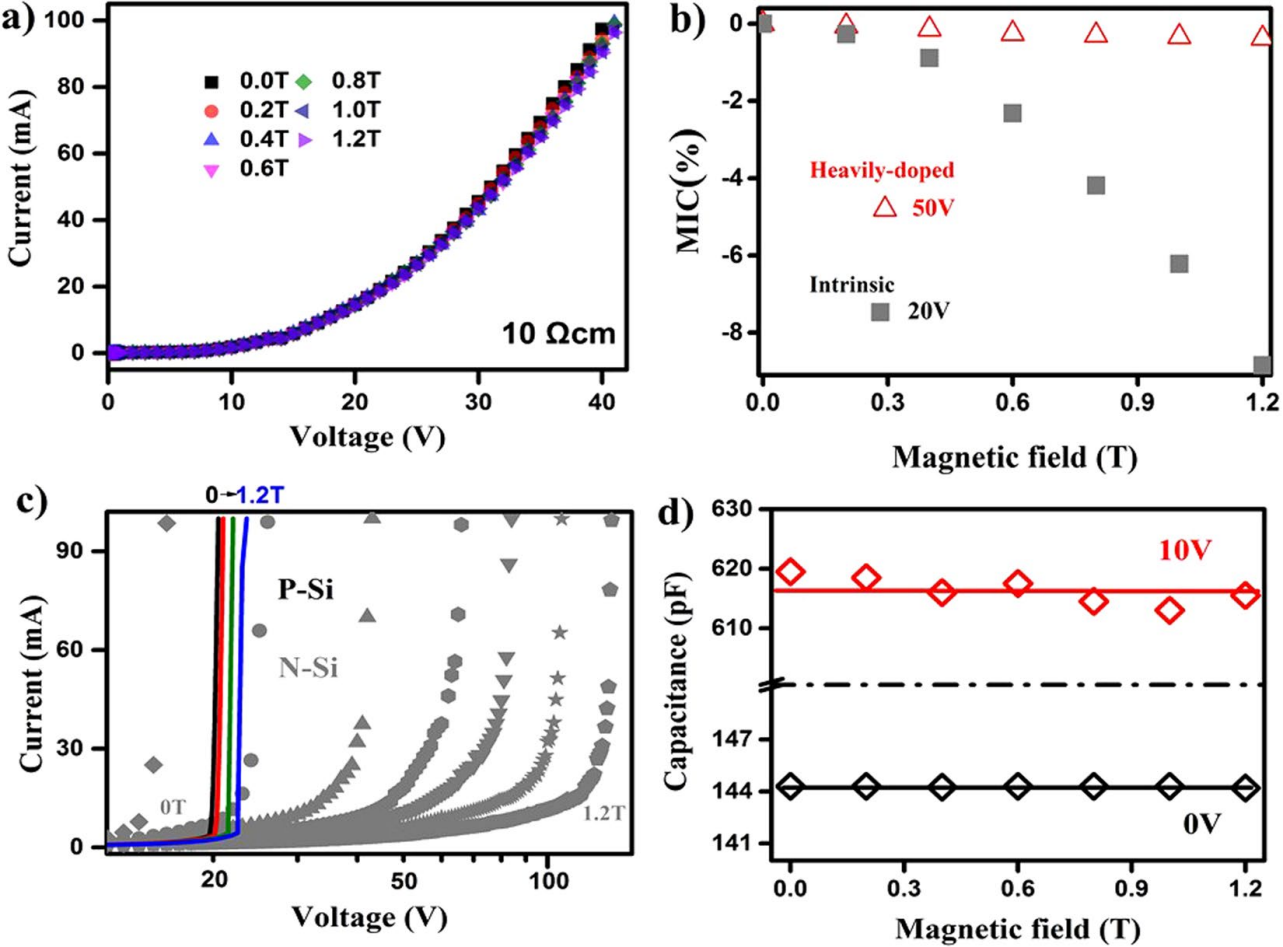
The *I-V* curves and the capacitance measurements of the heavily doped N-Si and the intrinsic P-Si. (**a**) The *I-V* curves for the heavily doped N-Si with nominal resistivity 10 Ω·*cm* are measured at room temperature under magnetic field ranging from 0 to 1.2 T. (**b**) *MIC* of a heavily-doped silicon has a 0.4% change from 0 to 1.2 T under 50 V bias voltage (open triangles) which is much smaller than the change of *MIC* of the intrinsic N-Si (grey solid squares). (**c**) The *I-V* curves for the intrinsic P-Si with nominal resistivity >1000 Ω·*cm* are measured at room temperature under magnetic field ranging from 0 to 1.2 T. In contrast to the significant variation of *V*_0_ in N-Si, there is a slight *V*_0_ shift in P-Si. The data of N-Si are re-plotted here for comparison. (**d**) Capacitances under different magnetic field measured at 0 and 10 V bias voltage are shown in black and red boxes, respectively.

In a heavily-doped semiconductor, the screening field destroys the inhomogeneity, and the huge magnetoresistance disappears^16^. The *I-V* curves of a heavily-doped N-Si under magnetic field ranging from 0 to 1.2 T are shown in Fig. 3a. There is an undetectable shift of *I-V* curves under magnetic field in both Ohmic and non-Ohmic regimes. *MIC* of a heavily-doped silicon only has a 0.4% change from 0 to 1.2 T under 50 V bias voltage, which is much smaller than the change of *MIC* in an intrinsic one, as presented in Fig. 3b. Obviously, the second order term of Hall and Lorentz deflection currents offset^21^, resulting in a negligible effect on the *I-V* curves and a small *MIC* change under magnetic fields in the heavily-doped N-Si. Altering the symmetry of the electrode set-up^14,17,31^ or producing doping density fluctuations^16,32^ purposely, huge linear magnetoresistances have been reported in heavily-doped N-Si. Utilizing the surface imperfection with an in-plane electrodes configuration, an asymmetric magnetoresistance response has also been realized in heavily-doped Ge^18^. In those methods, the electric-field-induced spatial dynamic of ionization and filling does not play a major role.

Few mechanisms are known to produce a large positive magnetoresistance in P-Si. Schoonus *et al*. obtained an extremely large magnetoresistance in a Si-SiO_2_-Al structure because the magnetic field raises the acceptor level^34^. Delmo *et al*. observed a large linear magnetoresistance in P-Si with an in-plane configuration due to the modulation of the electron-to-hole density ratio under magnetic fields^35^. By the symmetric out-of-plane electrode set-up, as illustrated in the inserted plot of Fig. 1a, we revisit the magnetoresistance for P-Si. Figure 3c shows the *I-V* curves under different magnetic fields as *θ* = 90° for an intrinsic P-Si. It is noted that the breakdown voltage only increases from 21 to 23 V between 0 and 1.2 T. Such phenomenon contracts to the intense magnetic-field variation of the breakdown voltages for an intrinsic N-Si, as demonstrated in the same plot by the gray dots. The relaxation of high-energy carriers depends on the intervalley scattering processes due to the different mobility of band valleys^36,37^. For silicon, the density states of heavy-hole band are much larger than that of light-hole and spin-orbit splitting bands^38^, resulting in weak intervalley scatterings and a small variation of hole mobility under electric fields. The spatial inhomogeneity of hole mobility in P-Si is trivial compared to that of electrons in N-Si. The magnetoresistance of P-Si is inconspicuous accordingly. Capacitances under different magnetic fields are presented in the Fig. 3d. The black and red boxes represent the capacitances measured at 0 and 10 V, respectively. The unchanged capacitances under magnetic fields confirm the uniform spatial distribution of the hole mobility in our intrinsic P-Si devices.

## Conclusion

In this letter, we reveal that under high electric field, the dynamic of ionization and filling arouses the strong electro-LO phonon interaction in N-type silicon, accompanied with the virtual phonon processes and the large lattice distortion. The dielectric response of silicon is thus a function of fields especially in non-equilibrium regimes, as evidenced by capacitance measurements. Further experiment and theory on carrier transport under the intense spatial charge dynamic are encouraged, which can shed light on applications in novel devices, ranging from energy-harvesting cells to novel magneto-electric devices.

## Electronic supplementary material

The space charge limited current and huge linear magnetoresistance in silicon
