# Supplementary material for: The space charge limited current and huge linear magnetoresistance in silicon
Source: Sci Rep. 2018 Jan 15;8:775. doi: 10.1038/s41598-017-19022-1 (PMC5768776; doi:10.1038/s41598-017-19022-1)
Supplement: Supplementary file 1 — The space charge limited current and huge linear magnetoresistance in silicon [file 41598_2017_19022_MOESM1_ESM.doc]

**The space charge limited current and huge linear magnetoresistance in silicon**

Y. Liu^1,2^, H. Wang*^1,2,3,4^ , X. Jin^1^ and M. Zhang^1,2^

^1^*Department of Physics, Capital Normal University*

^2^*Beijing Key Laboratory of Metamaterials and Devices*

^3^*Key Laboratory of Terahertz Optoelectronics, Ministry of Education,*

^4^*Beijing Advanced Innovation Center for Imaging Technology, Beijing, 100048, P.R. China*

**S1: The modified *Langevin* function: the expression of $\chi_{L}$.**

**S2: Parameters for fits to experiments.**

**S3: The discussions on the parameters** $\eta$ **and** $\gamma$

**S4: The dielectric constant under electric fields and magnetic fields**

**S5: The discussions on measurements.**

**S1: The modified Langevin function: the expression of** $\chi_{L}$

*Langevin* function $L(\alpha)$^1^: $\alpha=p_{0}E/\kappa_{B}T$, where $p_{0}$ is dipole moment under local electric field $E$ at temperature $T$. In Ohmic regime, free carrier drifts along electric field without spatial charge fluctuation. For non-polar silicon, the *Langevin* term is therefore ignored. But in space charge regime, the uncompensated charge and the concomitant push-back electrostatic field yield the spatial distribution of polarity-conserved charges. The localized charges are polarized along electric field, and the term of polarization convergence ought to be considered even for a non-polar one. We introduce a modified *Langevin* function in the framework of Mott-Gurney theorem to describes the polaron inhomogeneity under fields.

Under an assumption of field continuous model, $E$ is a function of the distance to electrode and can be expressed as: $E=\sqrt{\frac{8Jx}{9\mu_{n}\varepsilon_{0}\varepsilon_{r}}}$ by introducing Mott-Gurney law^2^. At any given local sites, the variation of current density originates from carrier density fluctuation due to the dynamic of ionization and filling. If neglecting the diffusion term under high electric field, the local current obeys $J\propto n$. Considering $E\propto\sqrt{\frac{8nx}{9\mu_{n}\varepsilon_{0}\varepsilon_{r}}}$ and the orientation polarizability $\frac{N_{p}p_{0}L(\alpha)}{\varepsilon_{0}E}$ in *Langevin* model, the polarizability $\chi_{L}$ in space charge regime can be expressed by:

$\chi_{L}=\frac{N_{p}p_{0}^{2}}{M\varepsilon_{0}\kappa_{B}T}\int_{n_{oh}}^{n_{\max}} \left(\frac{1}{3}-\frac{\beta^{2}n}{45}+\frac{2\beta^{4}n^{2}}{945}+...\right)dn$ (S3)

Where $\beta=\frac{p_{0}}{\kappa_{B}T}(\frac{8x}{9\mu_{n}\varepsilon_{0}\varepsilon_{r}})^{1/2}$. $N_{p}$ is the dipole density and $M$ is a partition function for the distribution of free carrier density. It needs to be addressed that the upper and the lower limits of the integral are $n_{oh}$ and $n_{\max}$ respectively. $n_{oh}$ is the carrier density in Ohmic regime referring to the thermal excited carriers, while $n_{\max}$ is the maximum localized carrier density originated from both the ionization and thermal carriers. $N_{p}p_{0}^{2}$ is a constant under an assumption of the uniform distribution of electromagnetic energy density along the transport direction. Finally, the analytical expression of $\chi_{L}$ can be expressed as $\gamma\ln(1+\Delta n/n_{0})$ with a coefficient $\gamma=\frac{np_{0}^{2}}{M\varepsilon_{0}\kappa_{B}T}$.

**S2: Parameters for fits to experiments**

**A. Intrinsic N-Si**

To fit the *I-V* curve of the intrinsic N-Si, the fixed parameters are the temperature *T*=300 K, the electrode distance *L*=0.5mm, and $\chi=11$. Due to the relatively large space distance between the doping atom, we ignore the tunneling current and set $\mu_{tD}=0$. The fitted parameters are the dopant concentration $N_{D}=1.2\times10^{17}m^{-3}$, the trap density $N_{D}=2.0\times10^{17}m^{-3}$, the donor energy level $E_{D}=E_{c}-0.3eV$and the trap energy level $E_{t}=E_{c}-0.49eV$, where $E_{c}$ is the energy level of the bottom of the conduction band. The electron effective mass, $m_{e}=0.2$. The acoustic-phonon mobility $\mu_{ac}=0.2$ at room temperature and the correction of parabolic band $A_{op}=10^{5}$.

**B. Heavily doped N-Si**

Due to the relatively small space distance between the doping atom, we set $\mu_{tD}=5\times10^{-5}$. The fitted parameters are the dopant concentration $N_{D}=3\times10^{20}m^{-3}$, the trap density $N_{t}=2\times10^{22}m^{-3}$, the donor energy level $E_{D}=E_{c}-0.4eV$ and the trap energy level $E_{t}=E_{c}-0.5eV$. The electron effective mass, $m_{e}=0.4$. The acoustic-phonon mobility $\mu_{ac}=8\times10^{-7}$ and $A_{op}=1$. The intrinsic polarizability,$\chi=8\times10^{6}$ selected for best fitting.

**S3: The discussions on the parameters** $\eta$ **and** $\gamma$

Fig.S1 Black and red hollow diamonds represent $\eta$ and $\gamma$, respectively, which are derived from I-V curves fitting. The solid lines are the curves guided by eyes to illustrate the relationship between $\eta$($\gamma$) and magnetic field, viz. $\eta\propto e^{-H}$ and $\gamma\propto H$.

The dynamic of ionization and filling is inevitably influenced by a perpendicular magnetic field with applied electric field, *viz*., prompting the traverse filling and suppressing the ionization along carrier transport direction. The successful simulations for those *I-V* curves are achieved by adjusting $\eta$ and $\gamma$ without modifying those material parameters, $N_{D}$, $N_{t}$ and energy levels etc. As illustrated in Fig.S1, $\eta$ decreases rapidly at low magnetic field and approaches to a constant as $H>0.4T$, which follows exponential decrease approximately, that is, $\eta\propto e^{-H}$. While the orientation coefficient $\gamma$ shows a monotonically decline, from 1.45 to -2, representing the suppression of $\chi_{L}$along the carrier motion and the expansion of the vertical direction.

**S4: The dielectric constant under electric fields and magnetic fields**

Fig.S2 The relationship between dielectric constant and voltage under magnetic fields.

The total dielectric constant $\varepsilon_{r-sum}$ is expressed as $1+\chi+\chi_{n}+\chi_{L}$, where$\chi$, $\chi_{n}$and $\chi_{L}$ are the intrinsic, the non-equilibrium and the orientation terms, respectively. $\chi$ is fixed as 11 according to literatures. $\chi_{n}$ is assumed proportional to the density of non-equilibrium carriers. $\chi_{n}\text{=}\eta\Delta n/n_{0}$, here $\Delta n$ defines as the density of non-equilibrium carriers, $n_{0}$ is the density of thermal-excited carriers and $\eta$ is a fitting parameter for simulation. $\chi_{L}$ is expressed as $\gamma\ln(1+\Delta n/n_{0})$ with a fitting coefficient $\gamma$. As shown in Fig.S2, $\varepsilon_{r-sum}$ approaches to a constant in Ohmic regime. At a critical voltage, $\varepsilon_{r-sum}$ shows a sharp, power-law rise with the increase of external voltage. $\varepsilon_{r-sum}$ is more sensitive to the magnetic field in the space-charge regime than in the Ohmic regime.

**S5: The discussions on measurements.**

In all electrical measurements, we used the high-precision multifunctional test platform Keithley 4200, which provides three measurement-speed modes that allow the user to select, i.e. fast, normal and quiet.


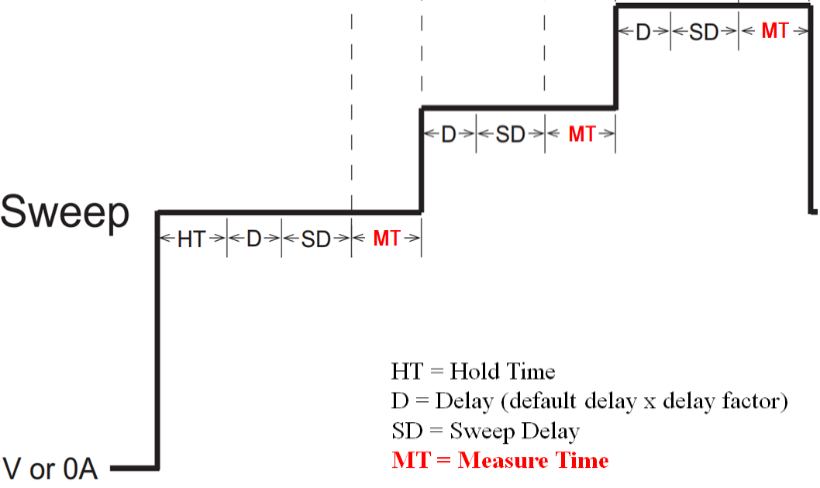


Fig.S3 The characteristic time intervals of a sweep measurement. [**Model 4200-SCS** **Reference Manual, 6-140**]

As shown in Fig.S3, a sweep measurement has four characteristic time intervals. Hold Time (HT): Note that at the start of each sweep there is a hold time. Delay (D): The delay time, allows the source to settle, and is measurement-range dependent. Sweep Delay (SD): The sweep delay provides additional settling time for each step in the sweep. It is a global setting. Measure Time (MT): The measure time is determined by the Filter Factor and the A/D Integration Time. D and SD are two crucial factors to control the different measurement speed.

*First, we present the measurement results by three basic measurement modes.*

We tested the same sample (*W*=16mm) in three different basic modes, as shown in Fig.S4(a). The results showed that the results measured in three models were highly repeatable. According to the manual, we know that these three test methods have different measurement intervals: Fast measurement speed mode: delay factor setting equals 0.7 and filter factor setting equals 0.2; Normal measurement speed mode: delay factor setting equals 1.0 and filter factor setting equals 1; Quiet measurement speed mode: delay factor setting equals 1.3 and filter factor setting equals 3. According to the manual [**Model 4200-SCS Reference Manual,6-135**], we know: Fast mode optimizes speed at the expense of noise performance; Normal mode provides a good combination of speed and low noise and is the best setting for most cases; Quiet mode optimizes low-noise measurements at the expense of speed. Therefore, the data shown in our manuscript were measured in Normal mode.

Fig.S4(a) The *I-V* curves under different measurement speed modes, *i.e.* Normal, Fast and Quiet exhibited as red, green and blue solid diamonds, respectively. (b) The *I-V* curves under different hold times, 0s (red hollow diamonds) and 20s (black down-triangles). (c) The *I-V* curves under different delay time, 0s (red hollow diamonds) and 5s (black down-triangles). (d) The comparison between the *I-V* curves measured by two different conditions, Normal sweep (red hollow diamonds) and a point by point method (black triangles).

Then, the influences of HT and SD under normal mode on measurements are presented in Fig.S4(b) and (c). We used a single sweep measurement, there is therefore no influence of HT on measurement reproducibility. Compare the curve obtained at SD = 0s, the data variations can be clearly seen as SD = 5s especially at high external electric fields. It is easy to understand that the thermal accumulation between two neighboring data affects the ionization and capturing processes. It is inevitable that the latter measurement state depends on the previous state, which universally exists in the process of non-equilibrium thermal electrons transport. In order to achieve an independent point by point measurement and check data reliability, we proposed a scheme for measurement, applying the voltage to certain values, deriving the last data in each *I-V* curves, drawing the collected data together in the same plot and finally forming a new *I-V* curve, as shown in Fig.S4(d). Although the time interval between two subsequent *I-V* curves is 10s, data are consistence with the one obtained in a single sweep condition. It proves again that the latter measurement state depends on the previous state and the experimental data given in our manuscript are objective physical facts. To achieve a theoretical fitting analysis on the repeatable data, all measurement data presented in our manuscript are collected under SD =0s in sweep condition. In addition, an in-plane electrodes configuration is mainly discussed in literatures with interesting results^3-6^. In contrast, a symmetric out-of-plane electrodes set-up with electrodes distance 500μm is used in our experiments. The shorter electrodes distance is helpful to suppress the influence of heat effect on charge dynamics.

*Secondly, we present the measurement results for different samples (with different size) by normal mode with HT =0s and SD = 0s.*

Sample size has influences on the measurement results. Two typical *I-V* curves with different lateral sizes, 4mm (open blue diamond) and 16mm (open red circles), are presented in Fig.S5(a). To reach a reasonable comparison, the geometric configuration of electrodes and the thickness of sample are fixed. In ohmic region, sample with large size has lower resistance, which is easily understood by a parallel resistance mode as the electrode size keeps the same value. As evidenced in Fig.S5(a), the slopes of *I-V* curves are quite similar in both sample in non-ohmic region. When approaching the breakdown voltage, the main conductive channel in the dielectric is determined by the filament effect. Although the lateral size of samples is different, the conductance in filament path dominants the overall resistance of sample (the same thickness of two samples, ~500μm). However, a significant resistance fluctuation is observed in samples with smaller lateral size in non-ohmic region. The joule heat depends on $I^{2}R$ and the thermal effect are quite similar in two samples. Samples with a smaller lateral size suffers a larger heat accumulation around electrode regions. The evidences are provided in Fig.S5(b) and (c), from which one can tell clearly, the indium electrode of the sample with *W*=16mm keeps its shape unchanged. That’s why, in our manuscript, we showed samples with a large size. In order to eliminate the minor factors as much as possible, realize the simulation and derive valuable information on local dynamics, only the results of samples with *W*=16mm are provided in our manuscript.

**(b)**

**(c)**


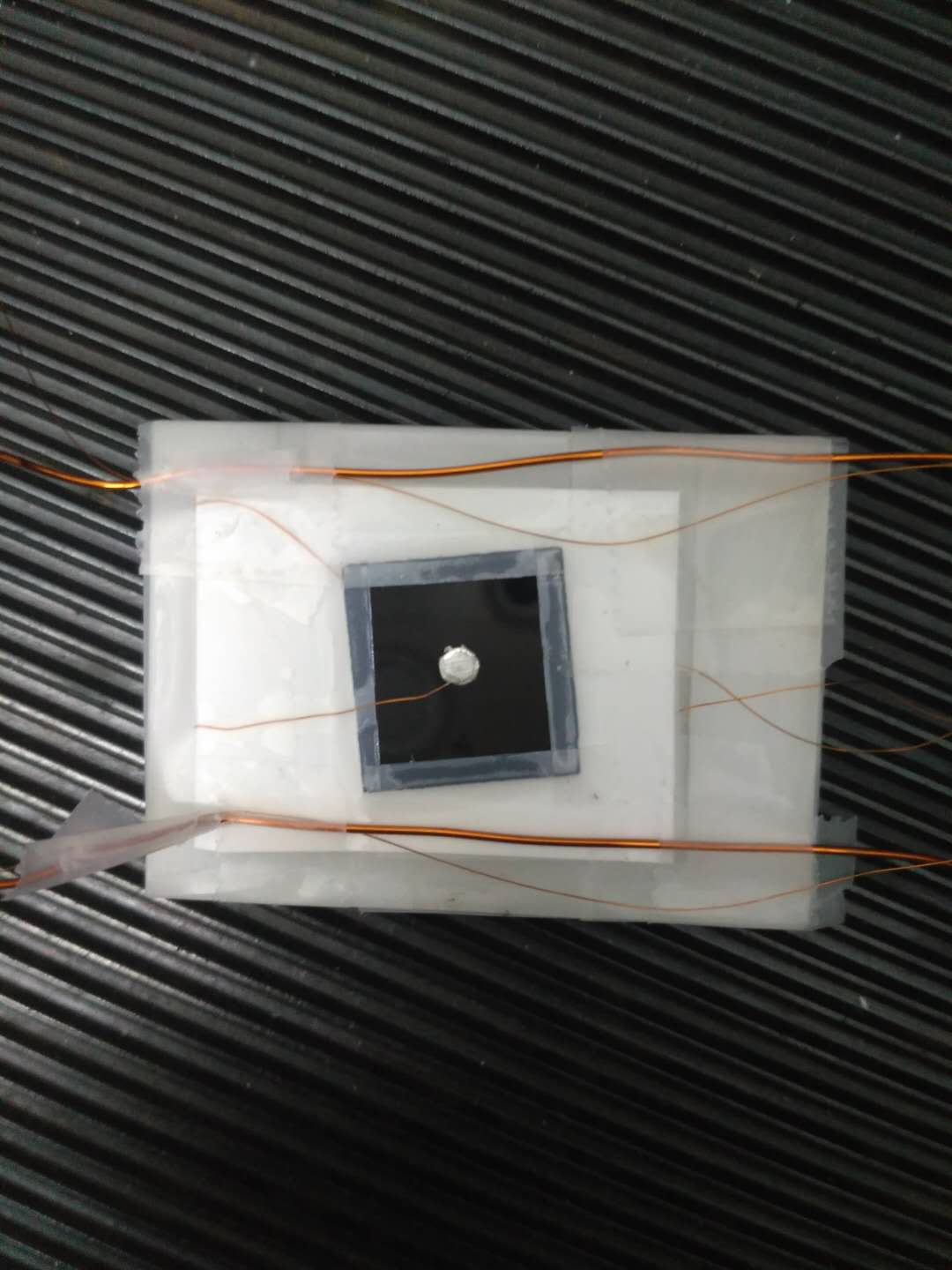

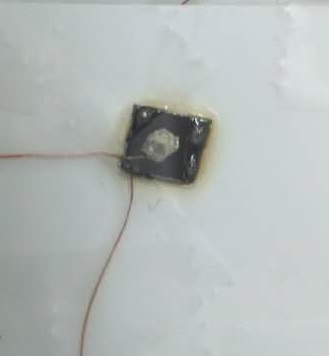


Fig.S5(a) The *I-V* curves for an intrinsic N-Si with nominal resistivity $>10K\Omega\cdot cm$with different lateral sizes, 4mm and 16mm, which shows in blue and red scatters, respectively. The photos of the sample after measurements are presented in (b) $4\times4\text{mm}^{2}$ and (c) $16\times16\text{mm}^{2}$.

*Thirdly, I-V curves measures in samples with W=8mm under different magnetic fields*

Fig.S6 The *I-V* curves of N-type silicon wafers under magnetic field. (a)10kΩcm, (b)1kΩcm, (c)10Ωcm and (d)0.1Ωcm.

Fig.S6 is the *I-V* curves of the samples with different doping concentrations acquired under the same measurement conditions: the electrode size ($1mm^{2}$), the sample thickness (500μm) and the lateral size ($8\times8mm^{2}$) at room temperature. The shift of *I-V* curve under magnetic field monotonically decreases with the increase of doping concentration, which is consistent with literatures^7^.

**References**

1. Feynman, R. P., Leighton, P. B. & Sands, M. *The Feynman's Lectures on Physics.* (Scientific & Technical Publishers, Shanghai, 2012).

2. Zhang, X.-G. & Pantelides, S. T. Theory of space charge limited currents. *Phys. Rev. Lett.* **108,** 266602 (2012).

3. Wan, C. H. *et al.* Nonlocal magnetoresistance due to Lorentz force in linear transport region in bulk silicon. *Appl. Phys. Lett.* **103,** 262406 (2013).

4. Wan, C. H., Zhang, X. Z., Gao, X. L., Wang, J. M. & Tan X. Y. Geometrical enhancement of low-field magnetoresistance in silicon. *nature* **477,** 304-307 (2011).

5. Chen, J. J. *et al.* Enhanced linear magnetoresistance of germanium at room temperature due to surface imperfection. *Appl. Phys. Lett.* **106,** 173503 (2015).

6. Solin, S. A., Thio, T., Hines, D. R., & Heremans J. J. Enhanced room-temperature geometric magnetoresistance in inhomogeneous narrow-gap semiconductors. *nature* **289,** 1530-1532 (2000).

7. Porter N. A. & Marrows C. H. Dependence of magnetoresistance on dopant density in phosphorous doped silicon. *J. Appl. Phys.* **109,** 07C703 (2011).
